# High-resolution Bayesian Virtual Epileptic Patient using neural field models

**DOI:** 10.1162/NETN.a.543

**Published:** 2026-04-22

**Authors:** Anirudh Nihalani Vattikonda, Meysam Hashemi, Marmaduke M. Woodman, Jean-Didier Lemarechal, Daniele Daini, Fabrice Bartolomei, Viktor Jirsa

**Affiliations:** Aix Marseille Univ, INSERM, INS, Inst Neurosci Syst, Marseille, France; Epileptology Department and Clinical Neurophysiology Department, Assistance publique des Hopitaux de Marseille, Marseille, France

**Keywords:** Focal epilepsy, Bayesian inference, Epileptor, Neural fields, Pseudospectral method

## Abstract

Epilepsy remains a significant medical challenge, particularly in drug-resistant cases where surgical intervention may be the only viable treatment option. Identifying the epileptogenic zone, the brain region responsible for seizure initiation, is a critical step in surgical planning. Combining dynamical system models, machine learning, and the neuroimaging data of epileptic patients in the so-called Bayesian Virtual Epileptic Patient (VEP) framework has previously been shown to be a promising approach for identifying the epileptogenic zone. However, previous studies employed coupled neural mass models to describe the whole-brain seizure dynamics and, hence, could only provide a highly coarse spatial estimate of the epileptogenic zone. In this study, we propose an extension of the Bayesian VEP to a neural field model, which can improve the spatial resolution by several orders. Performing model inversion using neural field models is a challenging task as the parameter space is very high dimensional, and it becomes computationally expensive to compute gradients. We demonstrate that by using pseudospectral methods and spherical harmonic transforms, it is feasible to perform model inversion on a neural field extension. We found that the high-resolution Bayesian VEP not only improves the spatial resolution but also significantly reduces the number of false positives.

## Author Summary

Accurate identification of the epileptogenic zone is critical for successful surgery in drug-resistant epilepsy. We present a high-resolution extension of the Bayesian Virtual Epileptic Patient framework—a multimodal approach that integrates personalized anatomical and functional data with neural field models to improve the estimation of the epileptogenic zone. By leveraging mathematical and machine learning techniques, we efficiently perform model inversion in high-dimensional settings. Validation on synthetic data and a retrospective patient cohort demonstrates the clinical potential of this approach.

## INTRODUCTION

Treatment for focal drug-resistant epilepsy typically involves surgical resection of brain regions, called Epileptogenic Zone (EZ; Bartolomei et al., 2017), which are hypothesized to be inducing seizures. However, in only about 60% of the cases are the patients seizure-free post surgery (Engel et al., 2003). Recently, several studies have proposed various methods to improve the presurgical estimation of EZ networks, which could thereby improve surgical outcomes (Cao et al., 2022; Jirsa et al., 2023; A. Li et al., 2021; Sohrabpour et al., 2020; Wang et al., 2023).

Commonly used metrics for presurgical evaluation include the Epileptogenicity Index (Bartolomei, Chauvel, & Wendling, 2008), which uses spectral and temporal information to define a quantitative measure of the degree of epileptogenicity of brain regions recorded through stereotactic EEG (SEEG). Epileptogenicity Maps (David et al., 2011) provide an anatomical map of seizure onset and propagation using spectral analysis of fast activity ranging from 60 to 100 Hz. Connectivity Epileptogenicity Index (Balatskaya et al., 2020) combines a directed connectivity-based measure with epileptogenicity index to improve EZ estimation in seizures with slow onset patterns. Epileptogenicity Rank (Parasuram, Gopinath, Pillai, Diwakar, & Kumar, 2021) is a modified epileptogenicity index measure that utilized temporal, spatial, and energy ratio during ictal discharges to estimate the EZ. These methods are purely data driven; that is, the EZ is estimated based on quantitative and/or statistical analysis of SEEG recordings of epileptic patients.

Alternatively, combining dynamical system models of epileptic seizures with Bayesian inference techniques has been demonstrated to be a promising approach to improve the presurgical estimation of the EZ (Cooray, Sengupta, Douglas, & Friston, 2016; Hashemi et al., 2020, 2021; Jafarian, Zeidman, Wykes, Walker, & Friston, 2021; Jha, Hashemi, Vattikonda, Wang, & Jirsa, 2022; Sip et al., 2021; Vattikonda et al., 2021; Wang et al., 2023). Such approaches mainly involve (a) defining a probabilistic model of seizure dynamics and (b) inferring parameters of the dynamical model using a Bayesian framework. Dynamical causal modeling (DCM; Friston, Harrison, & Penny, 2003) is a popular framework in neuroscience that demonstrated the usefulness of such a model-based approach in understanding various cognitive processes, such as vision (Adams, Aponte, Marshall, & Friston, 2015; Bastos et al., 2015), language (Ethofer et al., 2006; Yvert, Perrone-Bertolotti, Baciu, & David, 2012), and decision making (Stephan, Marshall, Penny, Friston, & Fink, 2007; Summerfield et al., 2006). In the context of epilepsy, DCM has been used to study the seizure propagation pathways from simultaneous EEG-fMRI data (Murta, Leal, Garrido, & Figueiredo, 2012), modulations in synaptic efficacy between regions during seizure onset (Papadopoulou et al., 2015), and concurrent changes in synaptic coupling at macro and meso-scales from calcium imaging data in zebrafish (Rosch, Hunter, Baldeweg, Friston, & Meyer, 2018).

DCM is primarily used to study the mechanisms of effective connectivity using statistical or biophysically plausible neuronal models. Bayesian inference over the Virtual Epileptic Patient (VEP; Hashemi et al., 2020; Jirsa et al., 2017) model proposed a similar framework to DCM, but principally aimed toward building personalized epilepsy models using a phenomenological model of epileptic seizure dynamics and advanced probabilistic machine learning techniques. Subsequent studies have demonstrated the efficacy of the VEP approach in identifying the EZ using point estimates (Penas et al., 2024; Vattikonda et al., 2021) and fully Bayesian posterior estimates (Hashemi et al., 2021; Jha et al., 2022) on a retrospective patient cohort. In VEP, the seizure dynamics are described using a phenomenological neural mass model (NMM) called epileptor (Jirsa, Stacey, Quilichini, Ivanov, & Bernard, 2014; Proix, Bartolomei, Chauvel, Bernard, & Jirsa, 2014). NMMs are simplified mathematical models used to describe the collective behavior of neurons within a certain brain region, rather than modeling individual neurons in detail. NMMs aggregate the activity of a large number of neurons into a smaller number of variables, making them computationally tractable for studying brain dynamics at a macroscopic level. Connectome-based brain network models describe the dynamics of the whole brain by considering the brain as a network of discrete interacting neuronal populations, where the interactions are typically constrained by the structural connectome (Deco, Jirsa, Robinson, Breakspear, & Friston, 2008; Honey et al., 2009) and the dynamics of each node are governed by an NMM. The whole-brain network models have been extensively used to understand the macroscopic brain dynamics in healthy (Breakspear, Heitmann, & Daffertshofer, 2010; Deco & Jirsa, 2012; Ghosh, Rho, McIntosh, Kötter, & Jirsa, 2008; Naskar, Vattikonda, Deco, Roy, & Banerjee, 2021; Yeo et al., 2011) and neurobiological underpinnings disease conditions (Bhattacharya, Coyle, & Maguire, 2011; Byrne, Coombes, & Liddle, 2019; Jirsa et al., 2023; Proix et al., 2014; Touboul, Wendling, Chauvel, & Faugeras, 2011; Wang et al., 2023). One of the key limitations of the previous studies is the highly coarse spatial resolution due to the simplification of whole-brain dynamics by the use of NMMs. In order to address this, here, we propose a neural field extension of VEP. While NMMs describe dynamics across time, neural field models (NFMs) describe the dynamics across space and time, allowing for infinite spatial resolution in theory (Jirsa & Haken, 1996). Moreover, compared with low-resolution NMMs (∼10*cm*^2^), high-resolution NFMs (∼1*mm*^2^) were shown to better explain empirically observed local field potential data (Hutt, Hashemi, & beim Graben, 2015; Jirsa, Jantzen, Fuchs, & Kelso, 2001, 2002; Pang et al., 2023; Pinotsis & Friston, 2014). It has recently been shown that the absence of local coupling in NMMs affects the estimation of excitability, which can have a significant clinical impact in the context of presurgical planning (Lemaréchal et al., 2024). However, several challenges exist in performing the Bayesian model inversion of NFMs at the whole-brain level: (a) they are computationally very expensive to simulate at the large-scale brain level; (b) model inversion requires computing gradients, which is computationally much more intensive than the simulation; and (c) dimensionality of the parameter space scales linearly with the spatial resolution.

Mapping the cortical surfaces onto a spherical surface and using a pseudospectral approach for numerical integration was recently shown to reduce the computational cost of neural field simulations by several orders of magnitude (Daini, Ceccarelli, Cataldo, & Jirsa, 2020; Daini & Jirsa, 2022). In this study, using the pseudospectral approach and reparameterizing the parameter space into spherical harmonic mode coefficients, we first demonstrate the plausibility of Bayesian model inversion of a neural field extension of the VEP model in order to estimate the EZ networks. To our best knowledge, this is the first study to demonstrate the Bayesian model inversion of a high-resolution NFM at the whole-brain level. Using synthetic data, robustness of the inversion across different resolutions and hyperparameters is systematically tested. Finally, we evaluate the accuracy of the NFM-based extension of the VEP model in estimating the EZ on a retrospective patient cohort and compare it with the previous NMM-based variant. We show that the neural field-based approach significantly improves the accuracy of EZ identification compared with the neural mass-based approach.

## METHODS

### Patient Data

To study the accuracy of the NFM-VEP model, it is tested against a retrospective patient cohort of 12 patients. The dataset used in this study is the same as the one used in previous VEP studies (Jha et al., 2022; Vattikonda et al., 2021; Wang et al., 2023). It includes invasive and noninvasive multimodal neuroimaging data such as T1-MRI, diffusion MRI, and SEEG recordings. For more details of the data acquisition process please refer to Wang et al. (2023). Informed written consent was obtained for all patients in compliance with the ethical requirements of the Declaration of Helsinki, and the study protocol was approved by the ethics committee—Comité de Protection des Personnes sud Méditerranée. For each patient with a known surgical outcome, a connectome is generated by running the reconstruction pipeline at high spatial resolutions, with the brain surface modeled as a triangular mesh containing 327,684 vertices (around 1 ∼*mm*^2^). Some of the patients are excluded due to issues with running the reconstruction pipeline at high spatial resolutions. This was mainly due to lesions or T1/DTI coregistration failures. Out of the 25 patients in the original dataset, the reconstruction pipeline ran successfully for 12 patients at high spatial resolution. These 12 patients are used for empirical validation in this study. Among the 12 patients, 7 of them are seizure-free post surgery, with their surgical outcome rated as Engel score I. In the remaining 5 patients, the surgery failed to achieve seizure freedom and their outcomes are classified as either Engel score II, III, or IV. More details of the patients such as age, gender, Engel scores, and inclusion/exclusion criteria are provided in Table 1. Patient IDs in Table 1 are not known to anyone outside the research group and do not reveal the identity of the patient to anyone outside the research group.

**Table 1.** Details of the retrospective patient dataset

**Patient ID**	**Gender**	**Age at epilepsy onset (y)**	**Epilepsy duration (y)**	**Epilepsy type**	**MRI**	**Hemisphere**	**Engel score**	**Inclusion**
id001	F	31–40	3	Temporo-insular	Normal	R	II	Yes
id003	M	21–30	13	Temporo-frontal	R temporo-occipital scar	R	I	Yes
id004	F	21–30	3	Temporal	R temporal mesial ganglioglioma	R	I	Yes
id007	M	51–60	5	Temporal	Normal	R > L	III	Yes
id008	F	31–40	8	Temporal	L amygdala enlargement	L	III	Yes
id009	F	11–20	34	Bifocal: parietal mesial and temporo-basal	Unknown R parietal lesion	R	III	No (lesion)
id010	F	21–30	18	Temporal	L hippocampal sclerosis	L	I	Yes
id011	F	21–30	14	Frontal	L frontal scar (abscess)	L	IV	No (lesion)
id013	M	1–10	17	Frontal	Normal	L	I	Yes
id014	F	1–10	18	Premotor	Normal	L	I	No (dwi/T1 coregistration failed)
id017	M	1–10	21	Temporal	L temporo-polar hypotrophy and hippocampal sclerosis	L	I	Yes
id020	F	11–20	10	Temporal	Normal	R	I	No (dwi/T1 coregistration failed)
id021	F	11–20	14	Occipital	Normal	L	II	No (dwi/T1 coregistration failed)
id022	F	1–10	23	Parietal	L parietal FCD	L	I	Yes
id025	M	1–10	35	Insular	Normal	L	I	No (dwi/T1 coregistration failed)
id027	M	11–20	12	Frontal	R prefrontal gliotic scar (AVM)	R > L	II	No (lesion)
id028	F	1–10	14	Temporo-frontal	Anterior temporal necrosis	R	III	No (lesion)
id030	M	11–20	45	Temporo-frontal	R frontal FCD	R	I	No (dwi/T1 coregistration failed)
id033	F	11–20	5	Temporo-parieto-opercular	Normal	R	IV	No (dwi/T1 coregistration failed)
id036	F	1–10	13	Temporo-insular	R temporal anterior resection cavity	R	IV	No (lesion)
id037	M	21–30	5	Temporal mesial	R temporo-polar & amygdala FCD, L postchiasmal pilocytic astrocytoma	R	III	No (dwi/T1 coregistration failed)
id039	M	41–50	4	Temporo-frontal	R fronto-temporal necrosis	R	I	Yes
id040	F	11–20	19	Temporal mesial	Hippocampal sclerosis	L	II	Yes
id045	F	21–30	17	Temporal mesial	Normal	L	IV	Yes
id050	M	21–30	21	Motor–premotor	L insulo-opercular necrosis	L	I	No (lesion)

Abbreviations: AVM = arteriovenous malformation; FCD = focal cortical dysplasia; L = left; R = right.

#### Connectome.

For each patient, using the reconstruction pipeline, a connectome is generated by parcellating the brain according to VEP atlas (Wang et al., 2021), consisting of 162 regions with 72 cortical and nine subcortical regions per hemisphere. Similar to other whole-brain studies using NMMs (Breakspear et al., 2010; Deco et al., 2008; Naskar et al., 2021), the VEP framework (Breakspear et al., 2010; Deco et al., 2008; Naskar et al., 2021) uses the connectome to define the long-range coupling between brain regions. In NMM models of whole-brain activity, the influence of one region on another is constrained by scaling the activity of a region through the connectivity. However, in this study, since an NFM is used, long-range coupling between two distinct regions is defined using the average activity of all vertices in a region, with the connectome constraining the influence of that region on others.

#### Forward model.

Neural activity at vertices is projected to the activity at the sensors using a so-called gain (or lead-field) matrix (Vattikonda et al., 2021; Wang et al., 2023), denoted by *G*. Assuming that the activity decays with a square of distance from the source, the gain coefficient mapping activity at source vertex *i* to sensor *j* is estimated as follows:

$$G_{ij}=\frac{a_{i}}{d_{ij}^{2}},$$ (1)

where *a*_*i*_ is the area of vertex *i* obtained by summing up one third of the area of all neighboring triangles. In this way, the gain matrix for the monopolar is constructed, and then the differences between the monopolar gains are taken to make the bipolar gain matrix, with additional corrections to ensure positivity. Matrix multiplication of the bipolar gain matrix and simulated source activity, given by the fast variable in the epileptor model (see the Dynamical Model section), yields the simulated SEEG signals. This procedure mirrors the empirical preprocessing of the SEEG data, where recordings were rereferenced to a bipolar montage obtained from the difference between neighboring contacts on the same electrode (Lachaux, Rudrauf, & Kahane, 2003). Hence, the forward model and the measured signals are expressed in the same reference frame, making the model variables directly comparable to the empirical bipolar SEEG signals. Note that, as for the scalp EEG, the choice of the reference and ground electrode is important in SEEG (Frauscher et al., 2024), and methods such as Laplacian referencing (G. Li et al., 2018) and ICA-based rereferencing can outperform conventional bipolar referencing in terms of sensitivity and specificity (Michelmann et al., 2018). Moreover, we have not taken into account the dependency of the source-to-sensor decay on the orientation of the neuronal tissue (a bias toward sensitivity over specificity). While the orientation plays an important role for the local field potential generated by the cortical tissue where a clear geometrical arrangement of the neurons exist (Buzsáki, Anastassiou, & Koch, 2012; Herreras, 2016), it is difficult to quantify this effect for the subcortical structures with their diverse structural arrangements. Thus, due to the lack of information about the orientation in subcortical structures (which would increase specificity but may reduce sensitivity, potentially missing true effects), we have chosen to omit the orientation dependency, as in previous studies (Jha et al., 2022; Vattikonda et al., 2021; Wang et al., 2023).

In this study, we use a pseudospectral method based upon spherical harmonics mode decomposition to efficiently compute numerical solutions of the NFM. The pseudospectral method is a general numerical scheme for solving partial differential equations, in which the solution is expanded in a set of basis functions (Fornberg & Sloan, 1994). A mode decomposition on the basis of spherical harmonics, representing smooth neural fields, has been used to reduce computational burden upon simulations of electro- and magneto-encephalogram (EEG/MEG) signals (Gabay & Robinson, 2017; Visser, Nicks, Faugeras, & Coombes, 2017; Vohryzek, Cabral, Vuust, Deco, & Kringelbach, 2022; Wingeier, Nunez, & Silberstein, 2001). Compared with finite-difference or finite-element discretizations, this approach achieves faster convergence with relatively few modes (Daini et al., 2020), making it well suited for modeling large-scale brain activity. Rather than evaluating derivatives directly, they are computed through the derivatives of the basis functions, which further improves efficiency. Thus, spherical harmonic transformation is particularly efficient in the context of high-resolution, whole-brain inference in epilepsy. Importantly, it aligns naturally with Laplacian decompositions on graph representations such as the connectome that has received significant attention in the literature for investigating structure–function relationships in the human brain (Aqil, Atasoy, Kringelbach, & Hindriks, 2021; Atasoy, Donnelly, & Pearson, 2016; Pang et al., 2023; Vohryzek, Sanz-Perl, Kringelbach, & Deco, 2025).

The pseudospectral method entails mapping the inflated spherical pial surfaces obtained from FreeSurfer (Fischl, Sereno, & Dale, 1999; Fischl, Sereno, Tootell, & Dale, 1999) to a regular grid on the spherical surface. The gain matrix corresponding to the regular spherical grid is estimated as the sum of the nearest vertices on the irregular grid, that is, as follows:

$${\hat{G}}_{ij}=\sum_{k\in D\left({\hat{v}}_{i}\right)}G_{kj},$$ (2)

where $D\left({\hat{v}}_{i}\right)$ represents all the vertices on the irregular spherical grid whose nearest neighbor is the vertex ${\hat{v}}_{i}$ on the regular spherical grid.

#### Resection masks.

Post surgical MRI is manually inspected to identify the brain regions in the (low-resolution) VEP parcellation that are resected by the surgery. A resection mask is created as a binary vector with as many elements as the number of regions in the VEP parcellation. The resection mask (*M*) is defined as follows:

$$M_{i}=\left(\begin{array}{cc} 1, & \text{if}\;\text{region}\;i\;\text{is}\;\text{resected},\\ 0, & \text{otherwise} \end{array}\right.$$ (3)

where *i* ∈ (1, 162) indexes one of the brain regions of the parcellation.

Since an NFM is used in this study, the model-predicted EZ are described at a high spatial resolution with 32,776 vertices per hemisphere. For comparison with the empirical outcomes, the resection masks at parcellation level (Equation 3) are mapped to a high resolution (*M^H^*) as follows:

$$M_{i}^{H}=\left(\begin{array}{cc} 1, & \text{if}\;M_{R\left(v_{i}\right)}\;\text{is}\;\text{resected},\\ 0, & \text{otherwise} \end{array}\right.$$ (4)

where, *i* ∈ (1, 65554), *v*_*i*_ represents vertex *i*, and *R*(*v*_*i*_) indicates the index of the brain region in the VEP parcellation that vertex *v*_*i*_ belongs to.

### Synthetic Data

To evaluate the accuracy of the EZ predictions by comparing them with ground truth, two synthetic datasets are generated using the connectome and gain matrices of the anonymous patients *id*004 and *id*022. EZs are defined by choosing regions close to the implanted electrodes to ensure identifiability (Hashemi et al., 2023). Specifically, for the patient *id*004, the EZ consisted of three regions: right-temporal-pole, right-T2-posterior and right-thalamus. The EZ for *id*022 consisted of four regions: left-superior-parietal-lobule-P1, left-intraparietal-sulcus, left-postcentral-gyrus, and left-postcentral-sulcus. SEEG electrode implantation, centers of the brain regions in the EZ, structural connectome, and the gain matrix of patient *id*004 and *id*022 are shown in Supporting Information Figure S1 and Figure S2, respectively. For all the vertices belonging to regions in the EZ, the tissue excitability parameter (denoted by *x*_0_) is set to −1.8 and for the rest of the vertices *x*_0_ = −3.0. Source activity for this spatial map of excitability is obtained by numerically solving the neural field extension of the 2D epileptor model (see Equation 6) using a pseudospectral method. Initial values of the fast (*x*) and slow (*z*) variables are set to −2.0 and 5.0, respectively.

### Feature Extraction

The raw SEEG data are preprocessed to extract SEEG log power. Preprocessing involves high-pass filtering raw SEEG data from 10 Hz, computing the power over a sliding window, applying a log transformation, and finally applying a low-pass filter of 0.05 Hz for smoothing out any short spikes in the data (Vattikonda et al., 2021; Wang et al., 2023). In order to reduce the computational cost of fitting, the SEEG log power time series is down-sampled to 300 time points. Data augmentation is a technique in machine learning to improve optimization when the observations are sparse. A common technique for data augmentation is to increase the number of observations by adding noise to the observed data. In this study, each time series is augmented by generating 50 more time series by adding Gaussian noise with mean zero and standard deviation of 0.1.

### Dynamical Model

Epileptor is a phenomenological model developed to describe the predominant dynamical characteristics of focal epileptic seizures (Jirsa et al., 2014). It is a general description of epileptic seizures that contains the complete taxonomy of system bifurcations to realistically reproduce the dynamics of onset, progression, and offset of seizure-like events (Saggio et al., 2020). Later, studies have extended the epileptor model to study the mechanisms of seizure recruitment and propagation through the whole brain using coupled NMMs (Proix et al., 2014) and NFMs (Proix, Jirsa, Bartolomei, Guye, & Truccolo, 2018). The full epileptor model consisted of five state variables with three distinct timescales: fast discharges (two variables), intermediate spike-and-wave events (two variables), and a slow variable accounting for extracellular effects and governing autonomous evolution between interictal and ictal phases (Jirsa et al., 2014). By taking advantage of timescale separation and focusing on the slower dynamics, the full epileptor model can be reduced to two variables (McIntosh & Jirsa, 2019; Proix et al., 2014). In a timescale separation, the fast discharge variables are assumed to quickly reach a quasi-steady-state or oscillate very rapidly compared with the other variables. Their aggregate behavior or “average” output is then captured by the new single “fast” variable. In essence, the intermediate timescale variables are not eliminated, but rather their rapid dynamics are averaged out or assumed to quickly follow the changes in the slower variables. The slow variable remains crucial for capturing the long-term transitions between different brain states (interictal, ictal, post-ictal). Hence, this reduction retains one slow variable consistent with the full model, and one fast variable, which can be interpreted as a proxy for transient changes in power dynamics between interictal and ictal phases (Hashemi et al., 2020, 2021; Vattikonda et al., 2021).

Previously, by embedding the 2D epileptor NMM into a Bayesian framework (Hashemi et al., 2020; Jha et al., 2022; Jirsa et al., 2017; Vattikonda et al., 2021; Wang et al., 2023), it was demonstrated that the model inversion could be performed to generate individualized models of seizure recruitment and propagation to improve presurgical identification of the EZ. In this study, we propose a neural field extension of the reduced epileptor model aimed toward improving the spatial resolution of the EZ predicted by the Bayesian VEP framework (Hashemi et al., 2020; Vattikonda et al., 2021). The dynamics of the 2D epileptor NFM at spatial position *y*_*i*_ and time *t* are defined as follows:

$$\partial_{t}x\left(y_{i},t\right)=1-x^{3}-2x^{2}-z+I$$ (5)

$$\partial_{t}z\left(y_{i},t\right)=\frac{1}{\tau }\left[4\left(x-x_{0}\right)-z-G\sum_{j=1}^{N_{n}}K_{ij}\left(x,(,\bar{y_{j}},),-,x,(,\bar{y_{j}},)\right)-\gamma \;W_{\mathrm{hom}}*S(x,\theta )\right]$$ (6)

where *i* and *j* index brain regions located at spatial coordinates *y*_*i*_ and *y*_*j*_, respectively, and *G* represents the global coupling, that is, a scaling factor for long-range connectivity *K*_*ij*_ between *i*−th and *j*−th regions, with *N*_*n*_ in total. The parameter *x*_0_ represents tissue excitability, and an isolated epileptor node produces seizure if *x*_0_ > −2.1. Moreover, $x(\bar{y_{j}})$ represents the average value of the state variable *x* in region *i*. Importantly, the term *W*_*hom*_^∗^*S*(*x*, *θ*) indicates the local or homogeneous coupling, with the operator^∗^ representing a spatial convolution, and it is defined as follows:

$$W_{\mathrm{hom}}*S\left(x,\theta \right)=\int_{-\infty }^{\infty }W_{\mathrm{hom}}\left(d\left(y_{i},y^{\prime }\right)\right)\;S\left(x,\theta \right)\;dy^{\prime }$$ (7)

where *d*(., .) is the distance along the surface between two points, *S* is a sigmoid function *S*(*q*, *θ*) = 1/(1 +*e*^−(*q*−*θ*)^), and *W*_*hom*_ is a short-ranged function representing local connectivity kernel typically defined as a Laplacian function *W*_*hom*_(*y*) = *e*^−∣*y*∣^/2. In this study, unless otherwise specified, *γ* = 1 and *θ* = −1. In computing numerical solutions of NFMs, the homogeneous connectivity was demonstrated to be a computational bottleneck (Daini & Jirsa, 2022). Performing model inversion entails computing gradients over the generated solutions. Typically, these gradients are computed using automatic differentiation (Baldy, Woodman, Jirsa, & Hashemi, 2025; Baydin, Pearlmutter, Radul, & Siskind, 2018), which builds a computational graph of all the operations or transformations involved in mapping a particular parameter configuration of the NFM (Equation 6) to the SEEG data features. Computationally intensive functions such as the homogeneous connectivity term in Equation 7 can lead to prohibitively high memory usage in computing gradients. To avoid such issues, we use a pseudospectral method in this study to estimate the homogeneous connectivity.

### Pseudospectral Method

The general dynamics of the neural field can be described by the following delayed integro-differential equations (Hashemi et al., 2025; Jirsa & Haken, 1996; Jirsa, 2009):

$$\begin{array}{l} \partial_{t}\psi \left(y,t\right)+N\left(\psi \left(y,t\right)\right)=\\ \int_{\Gamma }W_{\mathrm{hom}}\left(d_{g}\left(y,y^{\prime }\right)\right)S\left[\psi \left(y^{\prime },t-\frac{d_{g}\left(y,y^{\prime }\right)}{c}\right)\right]dy^{\prime }+\\ \int_{\Gamma }W_{het}\left(y,y^{\prime }\right)S\left[\psi \left(y^{\prime },t-\frac{d_{t}\left(y,y^{\prime }\right)}{v}\right)\right]dy^{\prime } \end{array}$$ (8)

where vector *ψ*(*y*, *t*) contains the state variables of the model (e.g., given the epileptor, *ψ*(*y*, *t*) = [*x*(*y*, *t*), *z*(*y*, *t*)])), measuring the local field potential of neural activity at time *t* and position *y*, in a general physical domain Γ, and function N representing the nonlinear contributions. This divides the brain connectivity into local (short-range; first term on the LHS of Equation 8) and global (long-range; second term on the left-hand side of Equation 8)) components, with *d*_*g*_ and *d*_*t*_ representing distance along the surface between two points and length of the fiber tracts connecting two points, respectively. The integral kernels *W*_*hom*_ and *W*_*het*_ are called the homogeneous (or short-range) and heterogeneous (or long-range) connectivity kernels, respectively, and are assumed to be known functions, while the parameters *c* and *v* denote the corresponding transmission speeds. Typically short-range cortical connectivity *W*_*hom*_ is assumed to rapidly decay with increasing distance and to be translationally invariant. Thus, a kernel is chosen and applied on the geodesic distances between points *y* and *y*′ on the cortex to obtain *W*_*hom*_. Common choices are Gaussian or Laplacian kernels (Daini & Jirsa, 2022; Hashemi et al., 2025; Jirsa, 2009). For *W*_*het*_ representing the long-range cortical connectivity, no general assumption is justified on a physiological basis (Jirsa & McIntosh, 2007). Assuming the heterogeneous pointwise connections, $W_{het}\left(y,y^{\prime }\right)=\sum_{i,j=1}^{N_{n}}K_{ij}\delta \left(y-y_{i}\right)\delta \left(y^{\prime }-y_{j}\right),\;i\neq j$, where *δ* is the Dirac Delta and *K*_*ij*_ represents the coupling strength through the myelinated connections. In this study, we considered instantaneous signal propagation (*c, v* → ∞).

It was shown that by mapping the high-resolution inflated surfaces obtained from FreeSurfer (Fischl, Sereno, & Dale, 1999; Fischl, Sereno, Tootell, & Dale, 1999) onto a unit sphere, neural field solutions can be obtained through a pseudospectral method (Daini & Jirsa, 2022), based upon mode decomposition on the spherical surface. Specifically, it was demonstrated that when the homogeneous connectivity kernel is a Laplacian function (exponential decay), a Taylor expansion in the linear domain of the sigmoid function and the symmetric properties of Spherical Harmonic Transforms (denoted by SHT) lead to the following:

$$W_{\mathrm{hom}}*\psi ≃N\psi +{SHT}^{-1}\left(-Dl\left(l+1\right)SHT\left(\psi \right)\right)$$ (9)

where *N* is a normalization coefficient and *D* is a diffusion coefficient. In approximating the homogeneous connectivity as given in Equation 9, using the fact that the homogeneous kernel (Laplacian here) is a short-ranged function and rotationally invariant, Daini and Jirsa (2022) found *N* and *D* to be the following:

$$N=r^{2}\int_{0}^{2\pi }\int_{0}^{\pi }\frac{1}{2}e^{-r\bar{\theta }}\text{sin}\left(\bar{\theta }\right)d\bar{\theta }d\phi$$

$$D=\frac{1}{4}r^{2}\int_{0}^{2\pi }\int_{0}^{\pi }{\bar{\theta }}^{2}\frac{1}{2}e^{-r\bar{\theta }}\text{sin}\left(\bar{\theta }\right)d\bar{\theta }d\phi$$

which for *r* = 100 mm are *N* ≃ 3.14128 and *D* ≃ 0.00047108. Computing the homogeneous connectivity using Equation 9 requires performing spherical harmonic transformation (Mohlenkamp, 1999; Wieczorek & Meschede, 2018). In this study, unless otherwise specified, the spherical harmonic mode coefficients are truncated up to a degree of 32 for computing the homogeneous connectivity.

#### Numerical solution.

To compute the trajectories of the neural field state variables governed by Equation 6, we performed numerical integration using Euler’s method with a time step of 0.05 s for a total of 600 steps. Since the SEEG data features are subsampled to contain 300 time points, the time series of the state variables obtained from numerical integration is subsampled at every 0.1 s.

### Bayesian Model Inversion

In the Bayesian VEP framework (Hashemi et al., 2020, 2023; Jha et al., 2022; Vattikonda et al., 2021; Wang et al., 2023), model inversion can be performed using either maximum a posteriori probability (MAP), variational inference, simulation-based inference, or Markov chain Monte Carlo (MCMC)-based sampling techniques over a posterior distribution:

$$P\left(\theta |D\right)\approx P\left(D|\theta \right)P\left(\theta \right)$$ (10)

where θ is a vector containing parameters of interest and *D* is the observed data features such as the SEEG log power. In this study, the parameter vector θ contains the initial values of the state variables (*x*(*y*, *t*_0_), *z*(*y*, *t*_0_)), spatial map of tissue excitability (*x*_0_(*y*)), global coupling (*G*), time scale parameter *τ*, observation noise (*ε*), and two scalar auxiliary parameters amplitude (*α*) and offset (*β*) defining a global linear transformation of the model predicted SEEG log power. In the spatial domain, with a spatial discretization of 32,768 vertices per cortical hemisphere, the dimensionality of the parameter space would be 196,667. In order to reduce the dimensionality of the parameter space, all the spatial maps are parameterized using SHT up to a degree of *L*_*max*_. In this study, we used *L*_*max*_ = 16 for representing spatial maps of *x*_0_ parameters, except while performing a parameter sweep across *L*_*max*_ for sensitivity analysis, reducing the dimensionality of the parameter space to 3,527.

Following Vattikonda et al. (2021), the prior distributions over the above parameters are defined as follows:

$$P\left(x\left(y_{i},t_{0}\right)\right)∼N\left(-3.0, 0.5\right)$$

$$P\left(z\left(y_{i},t_{0}\right)\right)∼N\left(5.0, 0.5\right)$$

$$P\left(x_{0}\left(y_{i}\right)\right)∼N\left(-3.0, 0.5\right)$$

$$P\left(G\right)∼N\left(1.0, 5.0\right)$$

$$P\left(\tau \right)∼U\left(20.0,100.0\right)$$

$$P\left(\varepsilon \right)∼N\left(0.1, 0.1\right)$$

$$P\left(\alpha \right)∼N\left(0.0, 1.0\right)$$

$$P\left(\beta \right)∼N\left(0.0, 1.0\right).$$

Taking advantage of prior knowledge of the parameter regimes of the dynamical model Equation 6 for seizure recruitment and propagation, all the priors with normal distributions are bounded above and below. The upper and lower bounds used in this study are provided in Table 2.

**Table 2.** Mean, variance, and upper and lower bounds for the inferred parameters

Parameter	Mean	Variance	Lower bound	Upper bound
*x*(*y*_*i*_, *t*_0_)	−3.0	0.5	−5.0	−1.5
*z*(*y*_*i*_, *t*_0_)	5.0	0.5	4.0	6.0
*x*_0_(*y*_*i*_)	−3.0	0.5	−5.0	0.0
*G*	1.0	5.0	0.0	10.0
*ε*	0.1	0.1	0.0	1.0
*α*	0.0	1.0	0.0	10.0
*β*	0.0	1.0	−10.0	10.0

In this study, by compromising on the full posterior calculation, model inversion is performed using MAP. To do this, an Adam optimizer (Kingma & Ba, 2014) is used to perform optimization with a learning rate of 0.001 and 5,000 steps as the termination criteria. Sensitivity analysis across different parameter sweeps is performed on CSCS high performance cluster where each node has the following configuration: Intel Xeon E5–2690 v3 @ 2.60 GHz (12 cores, 64 GB RAM) and NVIDIA Tesla P100 with 16 GB graphics memory. The rest of the analysis is performed on a workstation with Intel Core i9-10885H CPU @ 2.40 GHz (16 cores, 64 GB RAM) and NVIDIA Quadro RTX 4000 GPU with 8 GB graphics memory.

#### EZ estimation.

To classify the EZ from the inferred parameters, we used the seizure onset times similar to Vattikonda et al. (2021). Onset times are estimated by finding the time instant where depolarization shift occurred in the model-predicted activity of the fast variable (*x*). Next, all regions with onset times within a tolerance, *t_ε_*, of the earliest onset time, *t_λ_*, are classified as EZ. All other regions with onset times beyond *t_λ_* +*t_ε_* are classified as part of the propagation zone.

## RESULTS

### Validation on Synthetic Data

**Figure 1.**
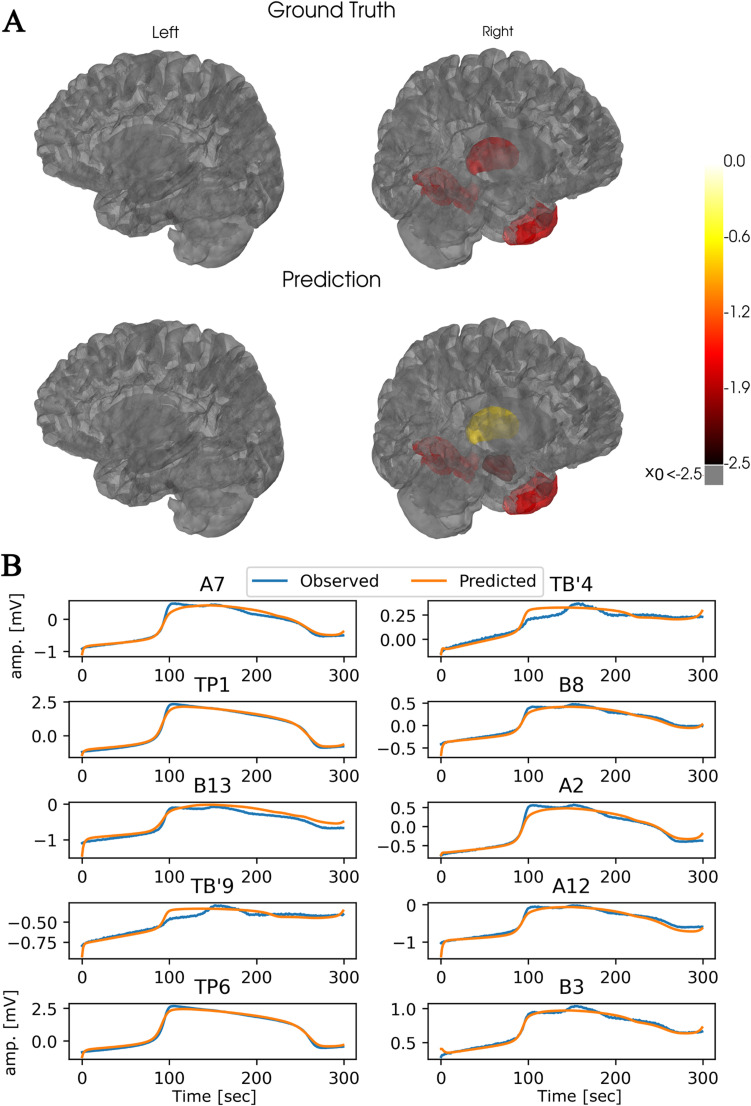
Inference accuracy on a synthetic dataset. (A) Comparison of the tissue excitability parameter (denoted by *x*_0_) between the ground truth synthetic data and the inferred values. (B) Goodness of fit between the observed and the predicted SEEG log power, for 10 randomly chosen sensors.

In order to compare the inferred EZ to the ground truth, first, the inference accuracy is tested against synthetic data. Figure 1A shows the tissue excitability parameter (denoted by *x*_0_) in ground truth and prediction. For details on fitting against synthetic data, see the Synthetic Data section and Supporting Information Figure S3 and Figure S4.

**Figure 2.**
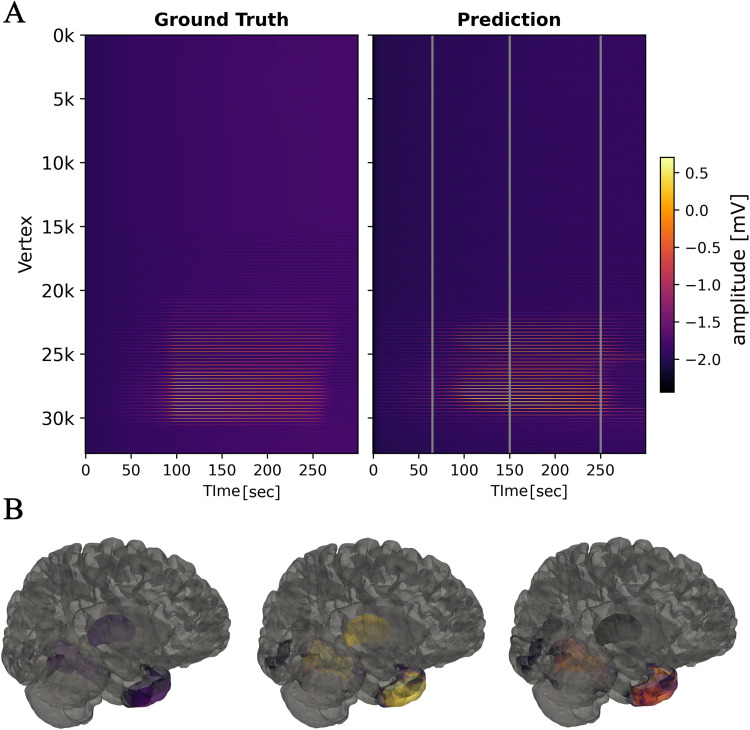
Comparison of source activity between ground truth and predictions. (A) Latent source activity obtained from the pseudospectral simulation of the neural field extension of the 2D epileptor model (left) and the source activity obtained from a simulation of the same model using the model parameters estimated by performing model inversion using MAP (right). (B) Inferred source activity at three different time steps, shown by gray vertical lines at the upper panel, illustrated on the brain surface. Color map is the same in both panels.

The results indicate that all three regions (right-temporal-pole, right-t2-posterior and right-thalamus) defined as part of the EZ in the ground truth are accurately inferred to have higher excitability than other regions. In addition to these three regions, one other region (right-T2-anterior) is inferred to have high excitability, even though the fit closely matches the observations at the sensor level, as shown in Figure 1B. However, as shown in Figure 2, this region is not recruited by the seizure at the latent source level. This demonstrates structural nonidentifiability, which arises from insufficient mapping between model states and observables. Nevertheless, this does not constitute practical nonidentifiability for the purpose of identifying the EZ, as it could still be uniquely determined from the trajectory of latent source states.

**Figure 3.**
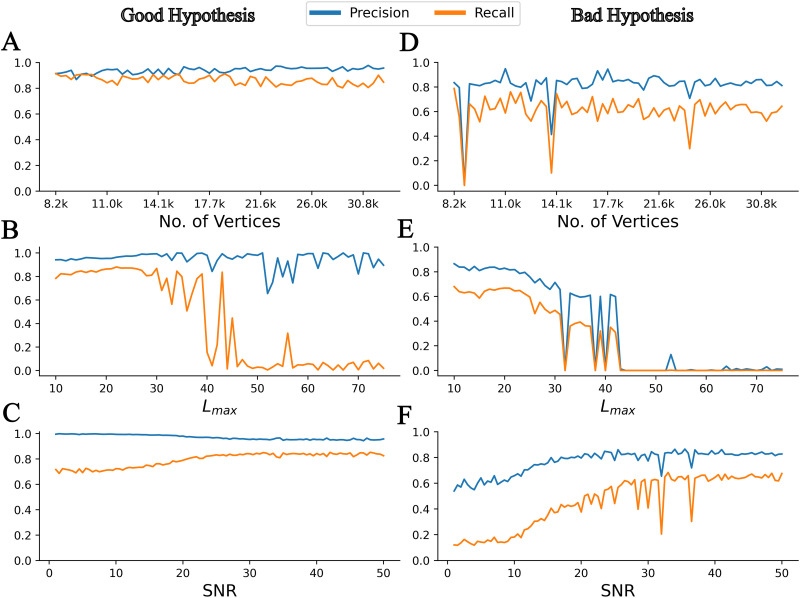
Sensitivity analysis across different hyperparameters of VEP with NFM (VEP-NFM) against synthetic data. Precision and recall of VEP-NFM model with a good EZ hypothesis across (A) different spatial resolutions, (B) different mode truncation parameters (*L*_*max*_), and (C) different signal-to-noise ratio (SNR) in the observed SEEG. (D, E, F) Same as the left panels, but with a bad EZ hypothesis.

Next, we tested the robustness of the inference using a pseudospectral method to changes in spatial resolution, mode truncation (see the Pseudospectral Method section), and observation noise. We found that the method is robust across different spatial resolutions but sensitive to mode truncation, as expected, and to observation noise. As shown in Figure 3A, both precision and recall of the inferred EZ remained above 80% across different spatial resolutions, ranging from 8,192 vertices to 32,768 vertices. Accuracy of the inferred EZ is found to decrease when higher spherical harmonic modes, that is, *L*_*max*_ > 25 are used to represent the spatial maps of parameters (see Figure 3B). This could be because higher number of modes have higher expressive power; that is, it can produce many distinct spatial maps, which in turn may lead to more local optima. Interestingly, this would imply lower truncations not only to help reduce the dimensionality of parameter space but also to reduce the structural nonidentifiability in the epileptor model by constraining the spatial maps of parameters to those that have smooth spatial transitions. However, such smooth spatial maps would have the trade-off that the size of the inferred EZ could be underestimated. To ensure that the effect of hyperparameters and observation noise on accuracy are not specific to the EZ hypothesis, the same parameter sweeps are performed using a bad EZ hypothesis (Figure 3E–F). Their effects are found to be similar to the case with a good EZ hypothesis (Figure 3A–C). A similar analysis performed on a different dataset is shown in Supporting Information Figure S5.

### Validation on Empirical Data

**Figure 4.**
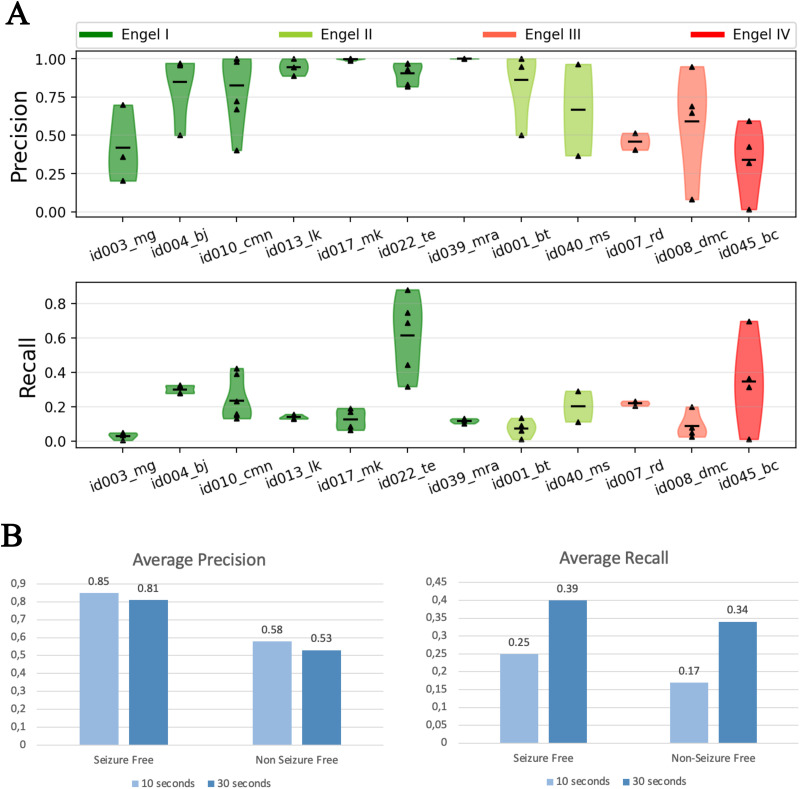
Analysis of accuracy of the predicted EZ in a retrospective patient cohort. (A) Distributions of precision and recall across seizures in each patient. Precision and recall are computed by comparing the predicted EZ against the EZ defined by postsurgical MRI and setting the onset tolerance threshold to 10 s. (B) Averaged precision and recall across seizures and patients, computed for different onset tolerance thresholds (10 and 30 s).

Accuracy of the predicted EZ in empirical data is validated using a retrospective patient cohort consisting of 12 patients (see Table 1). Out of the 12 patients, 7 patients are seizure-free post surgery and the remaining 5 are not seizure-free. Precision and recall of the predicted EZ compared with resection masks created from postsurgical MRI are shown in Figure 4A. In patients who are seizure-free post surgery (Engel score I), on average, precision and recall are 0.85 and 0.25, respectively. In patients who are not seizure-free post surgery, average precision and recall are 0.58 and 0.17, respectively. As evident from the precision, in patients where surgery was successful in achieving seizure freedom, the number of false positives are much less compared with patients who are not seizure-free post surgery. This would imply that the predicted EZ matches with the resected regions in patients where the surgery was successful but showed a mismatch in patients where surgery failed (see Supporting Information Figure S6).

Note that the low recall values do not necessarily imply that most of the resected regions are predicted to be healthy. We hypothesize this could be because: (a) some of the vertices classified as EZ in resection could be predicted as part of propagation zone based on onset tolerance threshold and (b) the resection masks are created by mapping low-resolution (162 nodes) parcellation masks to high resolution (65,554 vertices), but the predicted EZ, even if it includes all the distinct resected regions, may only include a fraction of the vertices from each region. The precision and recall, for each patient, with tolerance threshold to 10 s are shown in Figure 4A. Then, the averaged precision and recall of the predicted EZ, across seizures and patients, computed for different onset tolerance thresholds are shown in Figure 4B. Increasing the onset tolerance from 10 s to 30 s resulted in almost a twofold increase in recall. Average recall, per group, increased from 0.25 to 0.39 and 0.17 to 0.34 in seizure-free and non-seizure-free patients, respectively (Figure 4B, right), while average precision decreased slightly from 0.85 to 0.81 and 0.58 to 0.53 in seizure-free and non-seizure-free groups, respectively (Figure 4B, left).

**Figure 5.**
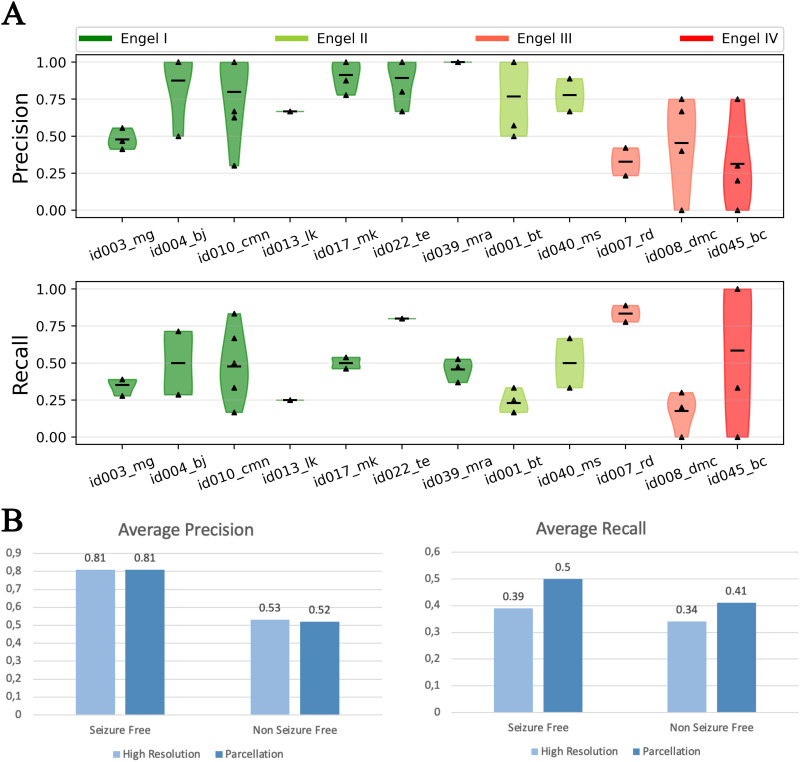
Analysis of the effect of mapping resection mask from VEP parcellation level to high resolution on inference accuracy, specifically on recall. (A) Similar to Figure 4A, except precision/recall are computed at the parcellation level. Resection masks at high resolution are constructed from the postsurgical resection masks available at the parcellation level. Its effect on recall is studied by mapping the predicted EZ to the parcellation level and computing precision and recall by comparing against resection masks at the parcellation level itself. (B) Averaged precision and recall across seizures and patients, computed for the high-resolution and (low-resolution) VEP parcellation level.

The effect of constructing high-resolution resection masks, from information at the low-resolution parcellation level, on recall can be investigated by computing the recall at the parcellation level. Predicted EZs at high resolution are mapped to parcellation level by thresholding on the percentage of vertices of a region in the predicted EZ. Accuracy of the predicted EZ in each patient when mapped to parcellation level is shown in Figure 5A. Considering regions with at least 20% of its vertices to be part of the EZ and at an onset tolerance of 30 s, average recall increased to 0.50 and 0.41 in seizure-free and non-seizure-free groups, respectively (Figure 5B, right). At the same thresholds, precision at the parcellation level is found to be almost the same as that observed at high resolution, 0.81 and 0.52 specifically (Figure 5B, left).

**Figure 6.**
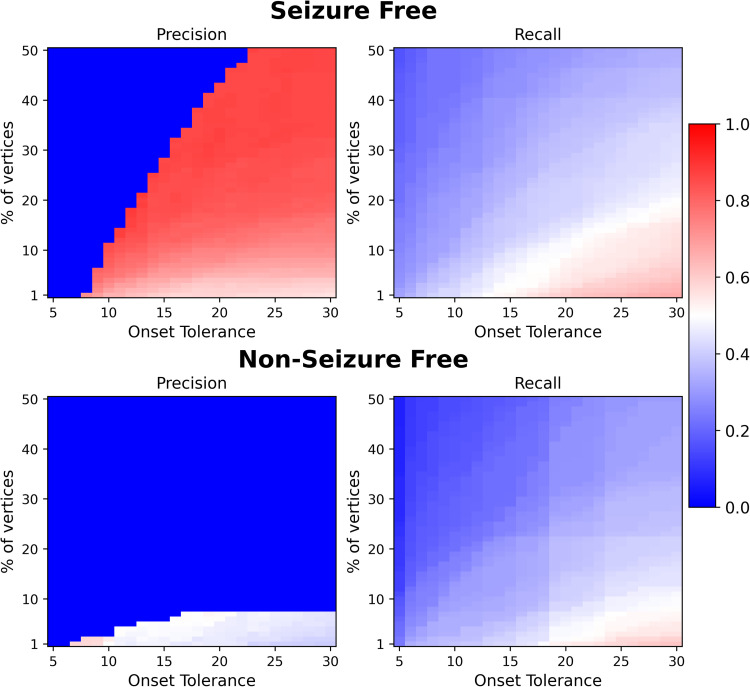
Analysis of effect of various thresholds performed in mapping the EZ to the parcellation level on prediction accuracy. The predicted EZ at high resolution is mapped to the parcellation level by thresholding on the minimum percentage of vertices in a region and the seizure onset time to be considered as part of the EZ. Average precision and recall, averaged across patients, across different values of these thresholds in a seizure-free group (top) and a non-seizure-free group (bottom). Note that in computing precision, there can be no regions in the EZ for low values of onset tolerance and high values of percentage of vertices. This can lead to division by zero errors; hence, if such an error occurred for any of the patients at a particular threshold, then the precision is set to zero.

The results observed in Figure 4 and Figure 5 provide evidence to our hypothesis that the reported low recall is a consequence of either thresholding or mapping information at the parcellation level to high resolutions. These effects are then summarized by performing a parameter sweep over both the thresholds of onset tolerance and percentage of vertices. Average precision and recall at the parcellation level across different thresholds are shown in Figure 6. As hypothesized, the recall increases as either onset tolerance is increased or by mapping the EZ to the parcellation level with lower fractions of vertices as inclusion criteria for the EZ.

**Figure 7.**
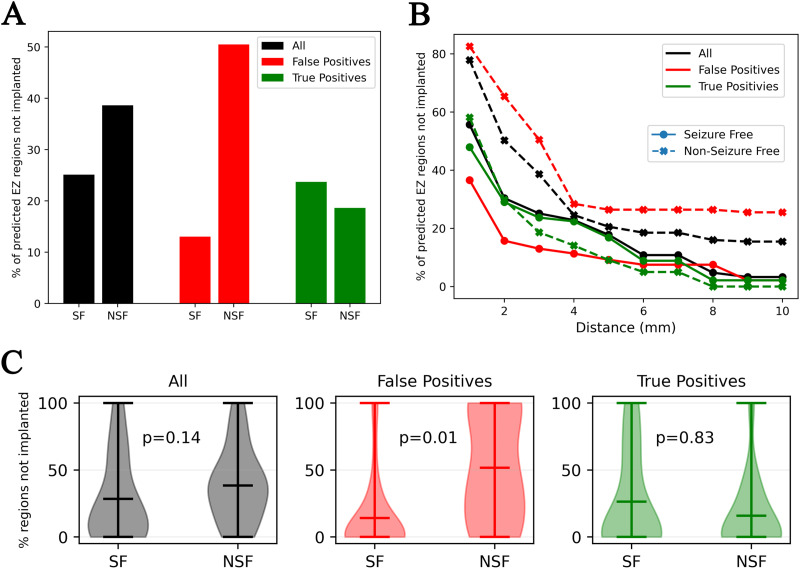
EZ discovery beyond implanted regions. (A) Comparison of percentage of regions, averaged across patients, beyond the implanted regions in seizure-free (denoted by SF) and non-seizure-free (denoted by NSF) groups. The tolerance for considering a region as implanted is set to 3 mm; that is, all regions within a radius of 3 mm of any implanted sensor are considered to be part of implanted regions. (B) Percentage of regions beyond implantation as the distance tolerance defining implantation is increased from 1 mm to 10 mm. (C) Distribution of percentage of regions not implanted across all patients and seizures with an implantation radius of 3 mm.

Identification of the seizure onset zone using purely data-driven signal analysis of SEEG time series such as Epileptogenicity Index, Epileptogenicity Maps, and Epileptogenicity Rank (Bartolomei et al., 2008; David et al., 2011; Parasuram et al., 2021) is, by construction, spatially constrained to identify the EZ within the implanted regions. Similar to previous VEP studies (Hashemi et al., 2020, 2023; Jirsa et al., 2017; Vattikonda et al., 2021; Wang et al., 2023), the generative model used in this study also includes a forward model, the so-called the gain matrix, transforming latent source activity to the measured SEEG time series. The forward model could account for seizure activity originating in regions beyond the implanted regions on the observed SEEG. The efficacy of the forward model in discovering the EZ beyond implanted regions is shown in Figure 7, by analyzing the number of regions in the predicted EZ that are beyond the implanted regions. Regions within a radius of 3 mm of any sensor are considered to be implanted. The number of regions, averaged across patients in each group, in the predicted EZ not part of the implantation is shown in Figure 7A. In the seizure-free group, 25% of the regions are found to be outside implantation while in the non-seizure-free group, it is 38%. Previous results (Figures 4 and 5) demonstrated that there are more false positives in the non-seizure-free group compared with the seizure-free group. Figure 7A also shows the percentage of predicted regions in the EZ that are outside implantation. Interestingly, among the false positives, the non-seizure-free group showed much higher regions outside implantation compared with the seizure-free group. Whereas, among the true positives, the difference is much smaller. It is reasonable to assume that among the non-seizure-free patients, the implantation missed some of the EZ. These findings provide confidence to that effect and demonstrate the efficacy of the proposed method in identifying the EZ beyond the implanted regions. However, under such rationale, it is also reasonable to hypothesize that: (a) among seizure-free patients, implantation should have covered the EZ, and (b) the true positives should be covered by the implantation irrespective of the group. Figure 7B shows the percentage of the predicted EZ regions not implanted, as the radius from the sensor is increased from 1 mm to 10 mm. As hypothesized, as the radius is increased, all the regions in the predicted EZ of seizure-free patients are covered by the implantation and, among the true positives, the percentage of regions outside implantation have reduced to less than 2% in both groups. The distributions of percentage of predicted regions in the EZ that are not implanted across all patients and seizures are shown in Figure 7C.

### Comparison to NMMs

**Figure 8.**
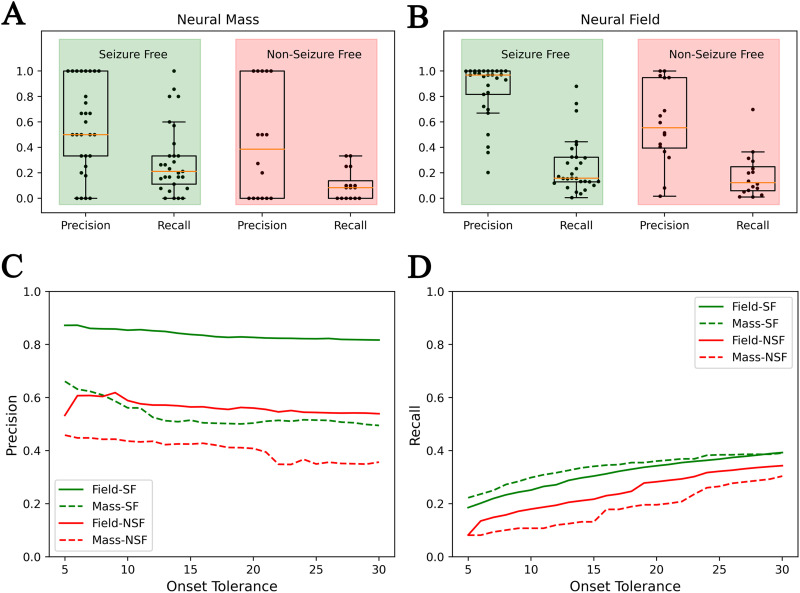
Comparison of prediction accuracy of VEP using a low-resolution neural mass versus a high-resolution neural fields model. (A) Precision and recall of VEP with a neural mass 2D epileptor model (VEP-NMM) against a retrospective dataset. (B) Precision and recall of VEP with a neural field 2D epileptor model (VEP-NFM) against the same retrospective dataset. (C, D) Precision and recall of VEP-NMM and VEP-NFM across various onset tolerance thresholds.

Lastly, the accuracy of the high-resolution neural field extension of the VEP (VEP-NFM) framework proposed in this study is compared against the previous VEP approaches with low-resolution NMM (VEP-NMM; Vattikonda et al., 2021; Wang et al., 2023) against the same retrospective dataset (see Figure 8). In the seizure-free group, at an onset tolerance of 10 s, the number of false positives have drastically reduced, as can be seen from the precision shown in Figure 8A and Figure 8B. A paired-samples *t* test was performed to compare precision and recall between VEP-NMM and VEP-NFM approaches. There is a statistically significant difference in precision between VEP-NFM (*M* = 0.85, *SD* = 0.21) and VEP-NMM (*M* = 0.56, *SD* = 0.34); *t*(28) = 5.41, *p* < 0.01. For recall, there is not a significant difference between VEP-NFM (*M* = 0.25, *SD* = 0.21) and VEP-NMM (*M* = 0.29, *SD* = 0.27); *t*(28) = −1.48, *p* = 0.15. In the non-seizure-free group, there is not a statistically significant difference between VEP-NMM and VEP-NFM in either precision or recall. At an onset tolerance of 30 s, precision is found to be statistically significant in both seizure-free and nonseizure groups, while recall is not. The average precision and recall in VEP-NMM and VEP-NFM, as the onset tolerance threshold is increased from 5 to 30 s, are shown in Figure 8C and Figure 8D. Finally, to assess the validity of the statistical analysis with our sample size (*N* = 12), we conducted a statistical power analysis for precision in the seizure-free group, using G*Power software (Faul, Erdfelder, Lang, & Buchner, 2007). For a significance level of 0.05, a statistical power of 0.8, and a sample size of 12, the minimum detectable effect size is 0.89. In our results in comparing the precision between VEP-NMM and VEP-NFM, the observed effect size is 1.06 and the achieved statistical power is 0.91, which is larger than the threshold.

## DISCUSSION

In this study, we explored the development of personalized models for the identification of the EZ by extending the VEP approach (Hashemi et al., 2020; Jha et al., 2022; Vattikonda et al., 2021; Wang et al., 2023) to the high-resolution NFM. Our primary objective was to enhance the spatial resolution of the predicted EZ using an NFM, which theoretically offers infinite spatial resolution. We employed a recently proposed pseudospectral method (Daini & Jirsa, 2022) to approximate solutions to the neural field equations, thereby avoiding the computational challenges associated with simulation and model inversion of NFMs.

Our findings demonstrate the success of our approach in achieving improved spatial resolution for EZ prediction. These findings could play an important role in the context of epilepsy research, as the accurate localization of the EZ is critical for guiding surgical interventions and improving patient outcomes. The use of NFMs proved to be a fruitful advancement, allowing us to harness their potential for high spatial precision. The adoption of the pseudospectral method reduced the computational cost of model inversion, providing a practical solution to overcome the computational hurdles that often accompany NFMs. This method presents a promising avenue for future research and may find applications in other brain disorders for diagnosis using Bayesian model inversion.

Systematic testing against synthetic data revealed that the prediction accuracy of our model remained robust against variations in spatial resolution, demonstrating its reliability in settings where data quality and resolution may vary. However, we noted a degradation in accuracy with higher degrees of spherical harmonics. We hypothesize this could be because a higher number of modes have higher expressive power, that is, they produce many distinct spatial maps that in turn would lead to more local optima. This observation prompts further investigation into the trade-offs between spatial resolution and computational efficiency in our approach. Strategies to mitigate this effect could be explored in future work. Moreover, the projection of brain surfaces onto a spherical mesh distorts distance relationships between vertices, leading to deviations from empirical spatial properties and inflated false positive rates (Bazinet, Liu, & Misic, 2025). Therefore, identifying a suitable orthogonal transformation that removes complex correlations in spatiotemporal data while preserving intrinsic spatial smoothness is essential. Recently developed techniques such as eigenstrapping (Koussis et al., 2025) offer a principled approach for rigorous statistical inference of cortical and subcortical associations, which remain to be considered in future studies.

The validation against retrospective data yielded encouraging results. Our model predictions exhibited a strong agreement with seizure-free patients, underscoring its potential as a tool for aiding in the diagnosis and treatment planning for epilepsy patients. Conversely, a notable mismatch was observed with non-seizure-free patients, highlighting the model’s potential for identifying individuals at a higher risk of recurrent seizures. These clinical insights demonstrate the practical applicability of our research and the potential for improved patient outcomes.

Given that the objective of this work was to establish and validate an efficient Bayesian framework on high-resolution epileptor neural field, this dataset is adequate for demonstrating proof of concept. Nevertheless, we acknowledge several limitations in our study. The retrospective dataset used for validation is small, and thereby validation against larger datasets is needed. Future research should consider more comprehensive datasets to enhance confidence of the proposed approach. Bayesian model inversion is performed using MAP, which provides point estimates using an optimization algorithm. Hence, the drawbacks of optimization algorithms such as getting stuck in a local minima also apply to our results. One common technique to partially mitigate this challenge is to run multiple optimizations with different initial conditions, which we did employ in this study. To address issues with MAP, we are currently exploring variational inference using more sophisticated density approximation methods such as Masked Auto-Regressive Flows (Papamakarios, Pavlakou, & Murray, 2017) and Free-Form Jacobian of Reversible Dynamics (Grathwohl, Chen, Bettencourt, Sutskever, & Duvenaud, 2018).

Moving forward, our research paves the way for several promising directions. Clinical validation through prospective studies is essential to further assess the reliability and real-world applicability of our models. We also anticipate opportunities for algorithm refinement, focusing on strategies to address the degradation in accuracy associated with higher degrees of spherical harmonics. Finally, the integration of our approach with other computational neuroscience techniques and complementary data sources could enhance the accuracy and clinical relevance of our personalized models.

In conclusion, our study advances the VEP framework by offering a novel approach to personalized models of epileptic seizure initiation and propagation. The use of NFMs and the pseudospectral method has shown promise in improving spatial resolution for EZ prediction, with clinical implications for diagnosis and treatment planning. While challenges and limitations remain, our research opens exciting avenues for future investigations.

## Acknowledgments

This research was supported by the Fondation pour la Recherche Médicale (Grant DIC20161236442), the European Union’s Horizon Europe Programme under the Specific Grant Agreement No. 101147319 (EBRAINS 2.0 Project), and the SATT Sud-Est (TVB-Epilepsy). This work has been carried out within the Fédération Hospitalo-Universitaire EPINEXT with the support of the Recherche Hospitalo-Universitaire EPINOV (Grant ANR-17-RHUS-0004) funded by the “Investissements d’Avenir” French Government program managed by the French National Research Agency (ANR). The authors also gratefully acknowledge the computing time granted through HBP-ICEI resources to access CSCS supercomputers in Switzerland.

## Supporting Information

The data that support the findings of this study are available on request from the corresponding authors. The data are not publicly available due to sensitive information that could compromise the privacy of research participants. Supporting information for this article is available at https://doi.org/10.1162/NETN.a.543.

## Author Contributions

Anirudh Nihalani Vattikonda: Conceptualization; Formal analysis; Investigation; Methodology; Software; Validation; Visualization; Writing – original draft; Writing – review & editing. Meysam Hashemi: Conceptualization; Investigation; Methodology; Software; Validation; Writing – original draft; Writing – review & editing. Marmaduke Woodman: Conceptualization; Methodology; Software. Jean-Didier Lemarechal: Data curation; Methodology; Software. Daniele Daini: Methodology; Software. Fabrice Bartolomei: Data curation; Resources. Viktor Jirsa: Conceptualization; Funding acquisition; Writing – review & editing.

## Data Availability

The patient datasets cannot be made publicly available due to the data protection concerns. The main code supporting this study is publicly available at https://github.com/ins-amu/infr_szr_prpgtn, *neural_fields* branch.

## Supplementary Material



## TECHNICAL TERMS

Epileptogenic Zone (EZ): The connected brain regions responsible for the primary organization and initiation of seizures. Sometimes, this is described as an area of brain regions that is necessary and sufficient for the initiation of seizures, such that its removal guarantees complete seizure abolition.
Bayesian inference: A principled method for statistical inference that updates prior (initial) beliefs about a hypothesis using new evidence (from observed data), thereby naturally characterizing uncertainty over unknown quantities.
Virtual Epileptic Patient (VEP): It is a workflow that integrates personalized whole-brain models, patient-specific anatomical and functional data, and machine learning methods to support clinical decision-making by estimating the epileptogenic zones.
Neural mass models (NMMs): A simplified mathematical modeling approach that represents the activity of large populations of strongly interconnected neurons as a single entity, using differential equations to describe their collective dynamics at the mesoscopic scale.
Epileptor: A phenomenological model based on a system of coupled nonlinear differential equations generating epileptic dynamics.
Neural field models (NFMs): A mathematical framework that models the continuous spatial and temporal dynamics of neuronal activity using partial differential equations to capture the propagation of activity across the cortical surface.
Pseudospectral method: A numerical technique for solving partial differential equations by transforming the problem into simpler spectral space for efficient and accurate computation, typically using global basis functions such as Fourier or spherical harmonics.
Spherical harmonic transforms: A mathematical technique that represents functions defined on the surface of a sphere as a weighted sum of spherical harmonic basis functions, enabling the casting of spatial data to the spectral domain and vice versa.
Maximum a posteriori probability (MAP): An estimation method in Bayesian inference that selects the value of an unknown quantity that maximizes the posterior probability, given the observed data.
