# Supplementary material for: High-resolution Bayesian Virtual Epileptic Patient using neural field models
Source: Netw Neurosci. 2026 Apr 22;10(2):374–99. doi: 10.1162/NETN.a.543 (PMC13108505; doi:10.1162/NETN.a.543)
Supplement: Supplementary file 1 [file netn-10-2-374-s001.pdf]

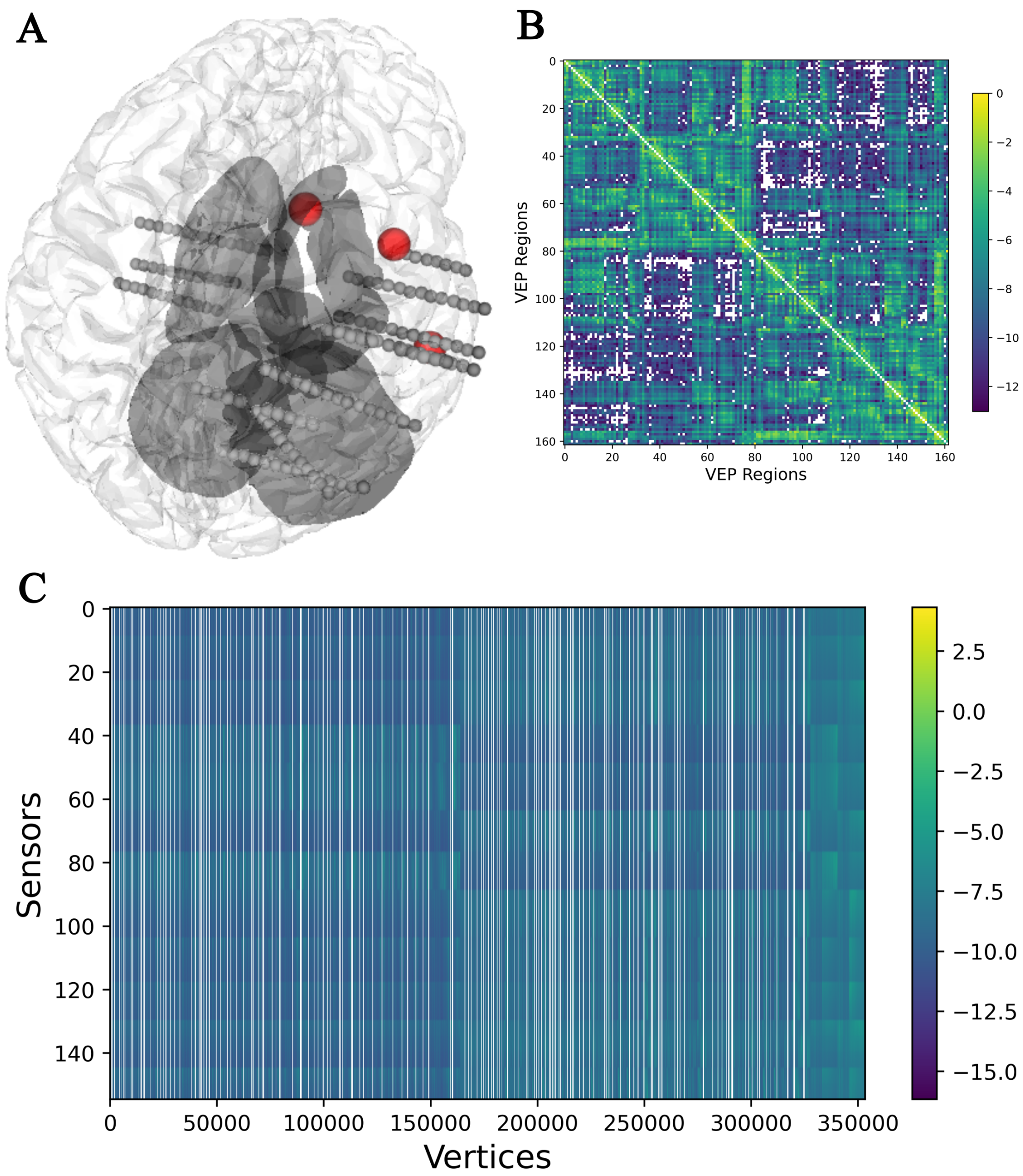

Figure S1: Details of patient *id*004 used for generating synthetic data. (**A**) SEEG electrode implantation. Sensors are shown in gray solid balls and centers of the brain regions in Epileptogenic Zone are shown in red balls. (**B**) Structural connectome constructed from diffusion MRI. (**C**) Gain matrix.

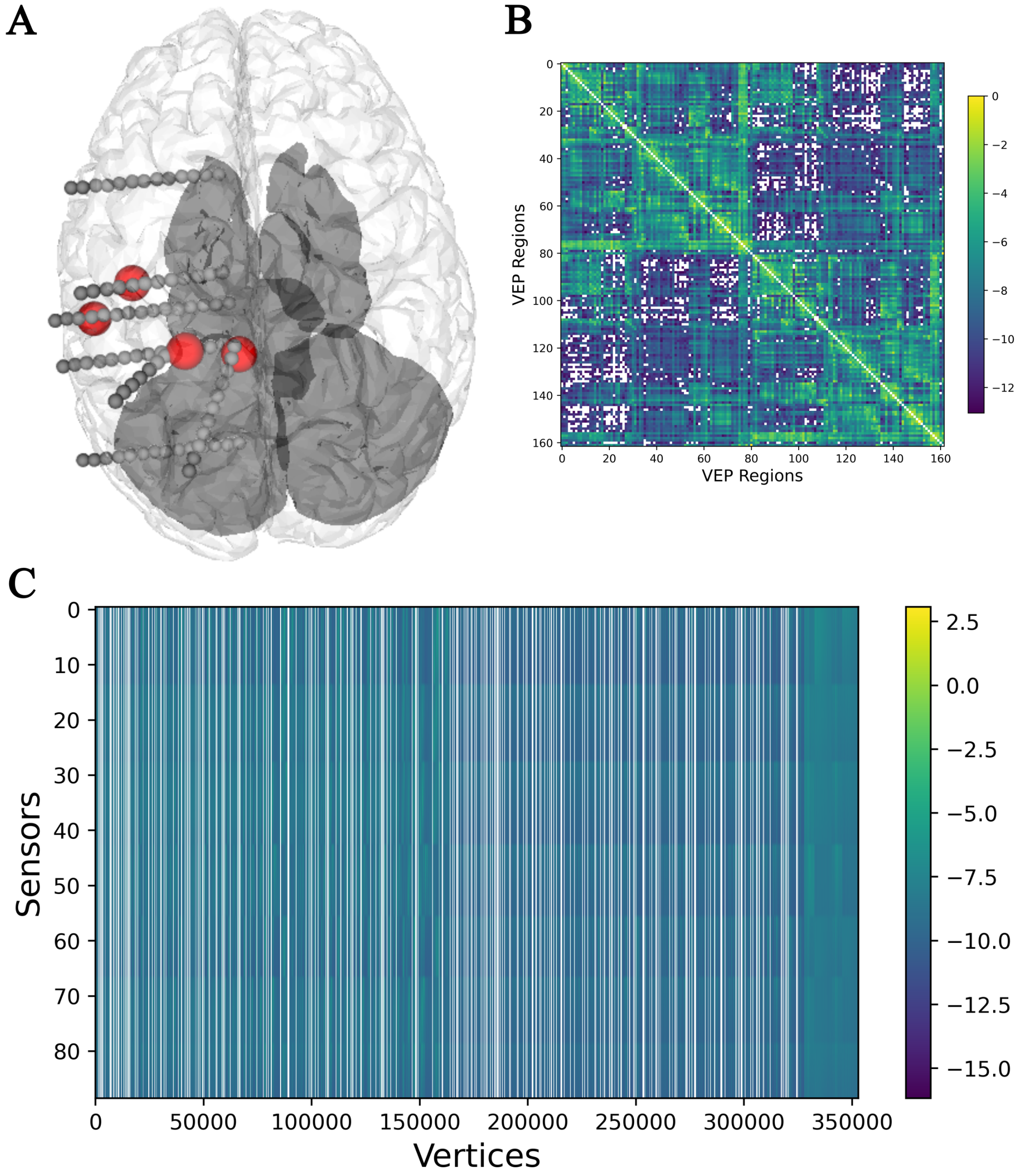

Figure S2: Details of patient $id022$ used for generating synthetic data. (**A**) SEEG electrode implantation. Sensors are shown in gray solid balls and centers of the brain regions in Epileptogenic Zone are shown in red balls. (**B**) Structural connectome constructed from diffusion MRI. (**C**) Gain matrix.

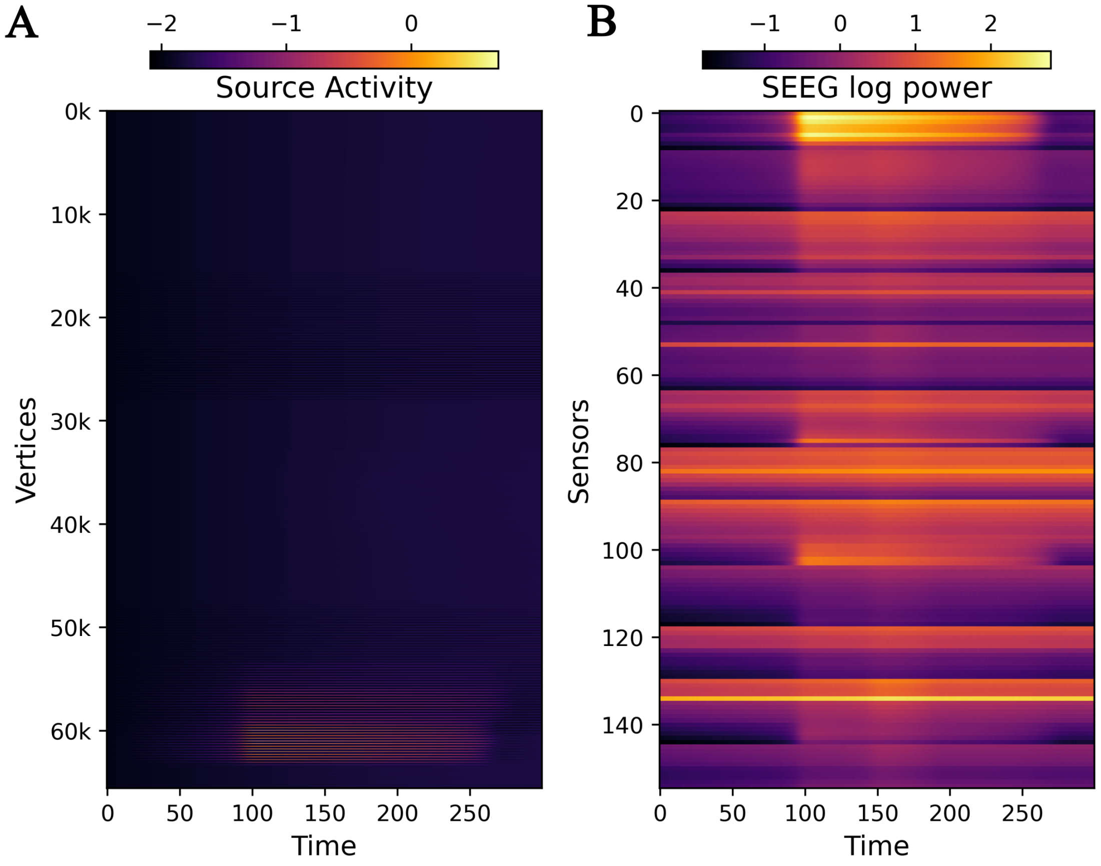

Figure S3: Synthetic data generated based on data of patient *id*004. (**A**) Simulated source activity. (**B**) Observed SEEG log power obtained by projecting the simulated source activity in panel **A**, using the gain matrix shown in S1.

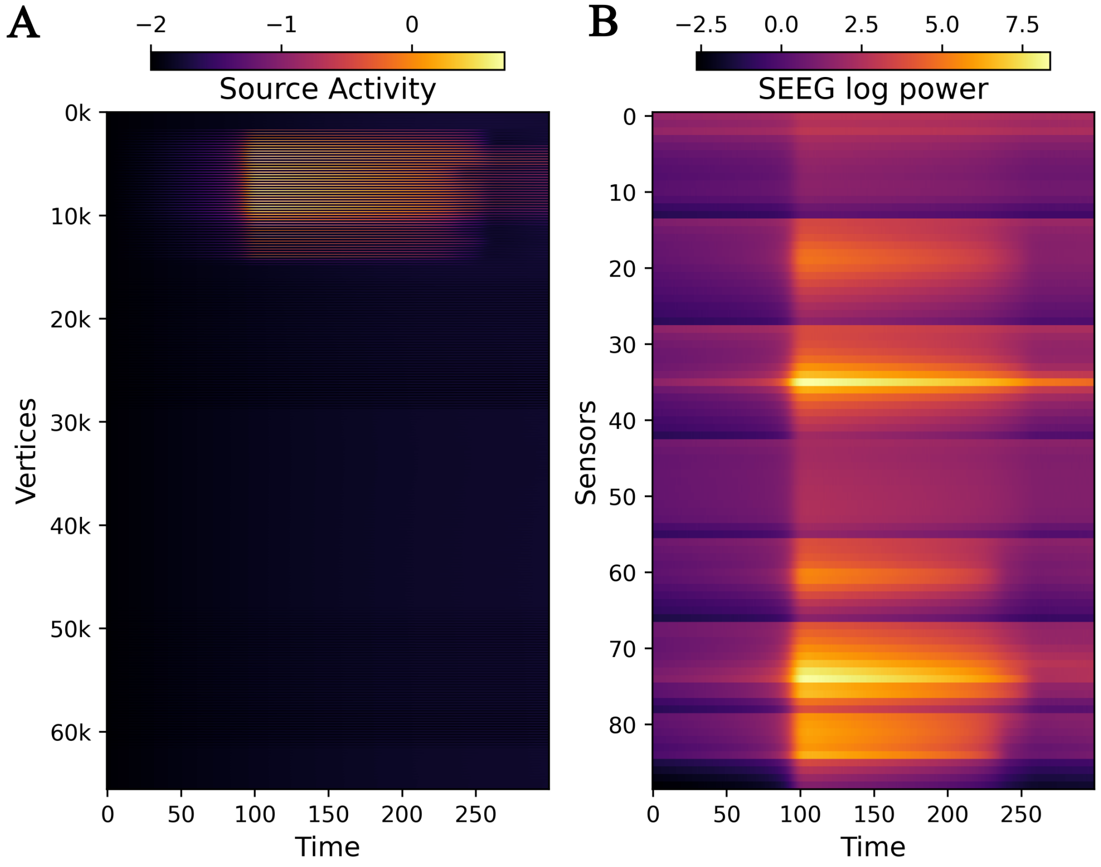

Figure S4: Synthetic data generated based on data of patient *id*022. (**A**) Simulated source activity. (**B**) Observed SEEG log power obtained by projecting the simulated source activity in panel **A**, using the gain matrix shown in S2.

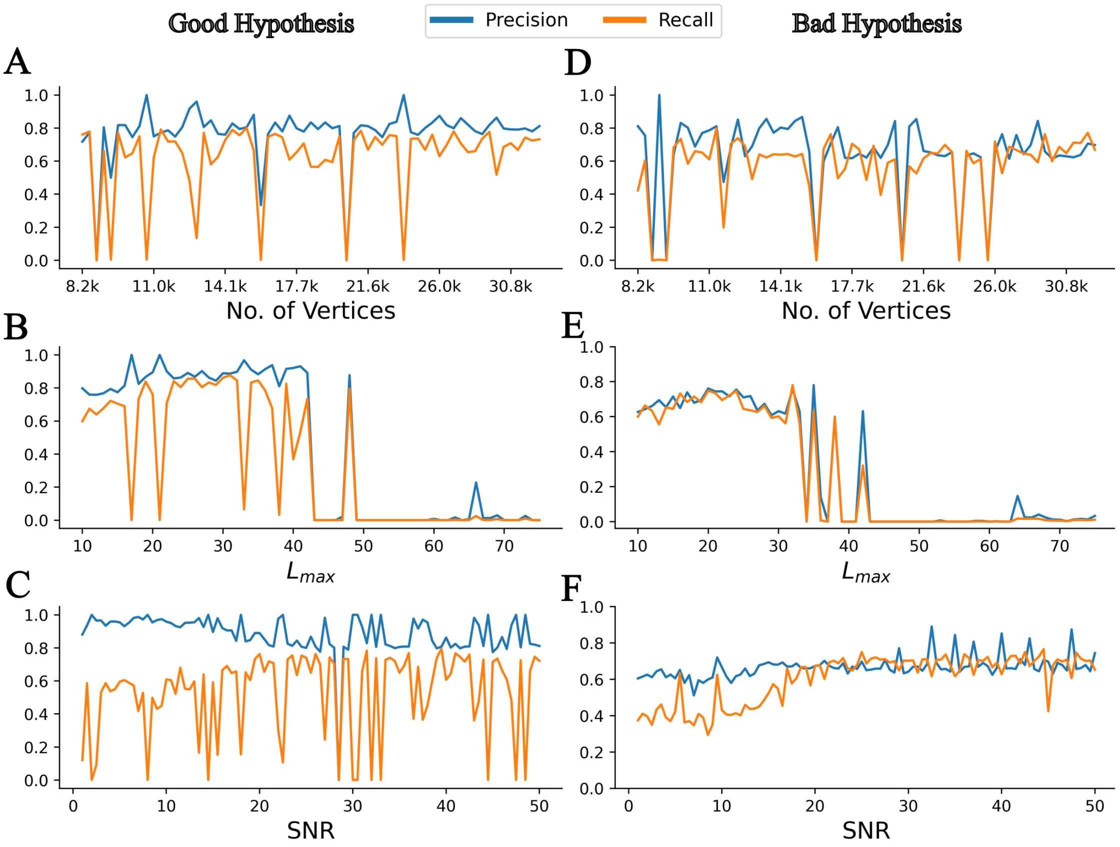

Figure S5: Sensitivity analysis across different hyper-parameters of VEP with neural field model (VEP-NFM) against synthetic data using data of patient *id*004. Precision and recall of VEP-NFM model with a good EZ hypothesis across (**A**) different spatial resolutions, (**A**) different mode truncation parameters (L_{max}), and (**C**) different signal to noise ratio (SNR) in the observed SEEG. (**D**, **E**, **F**) Same as the left panels, but with a bad EZ hypothesis.

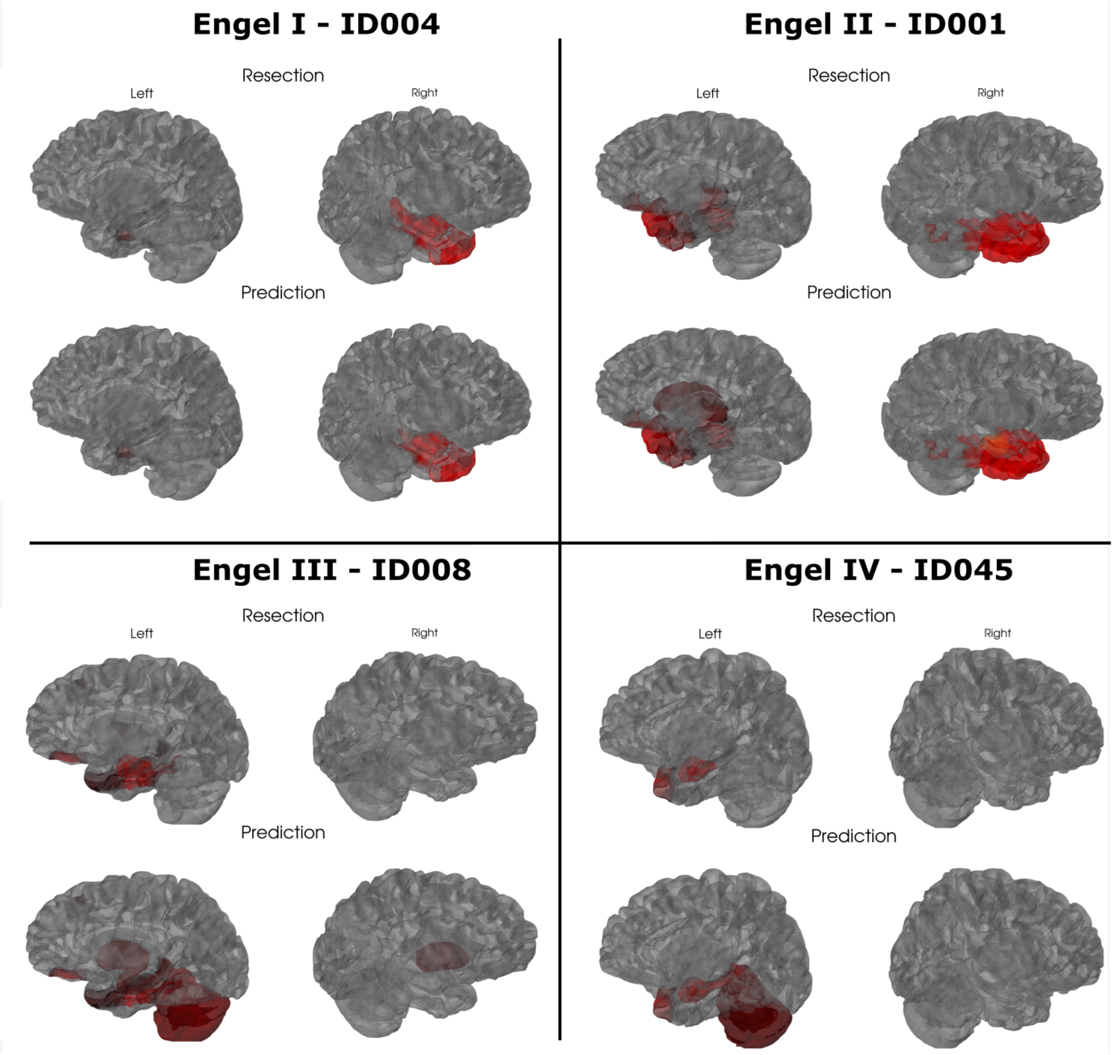

Figure S6: Comparison of resected regions with predicted EZ of four patients with various Engel Scores. Note that since resection information was only available at parcellation level, it is mapped to high resolution by considering all vertices within a resected region as part of the resection.
